# Supplementary figures and images for: Fine-Scale Recombination Maps of Fungal Plant Pathogens Reveal Dynamic Recombination Landscapes and Intragenic Hotspots
Source: Genetics. 2017 Dec 20;208(3):1209–29. doi: 10.1534/genetics.117.300502 (PMC5844332; doi:10.1534/genetics.117.300502)

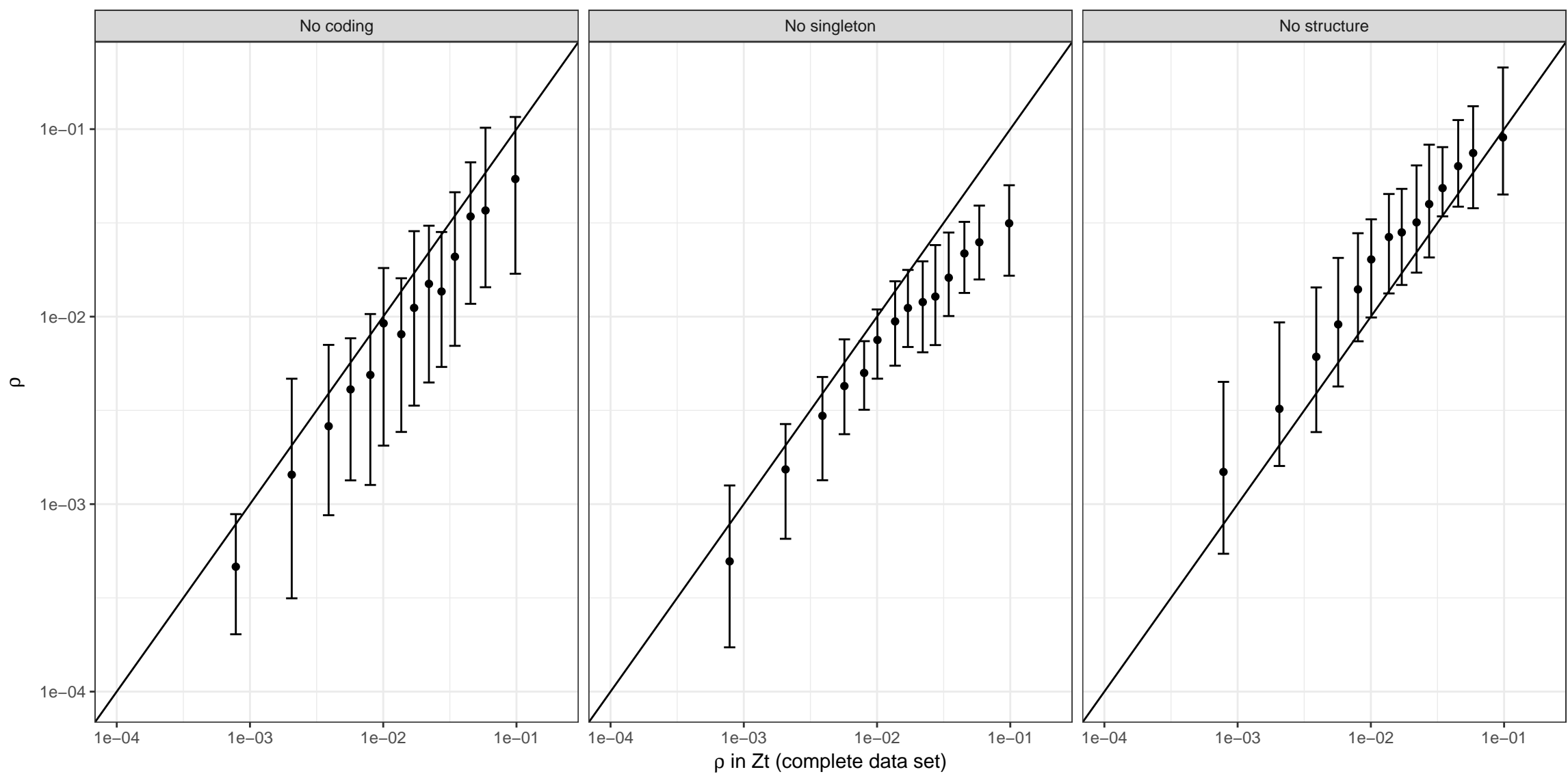

Supplement: Supplementary file 1 [file 1209FigureS1.pdf]

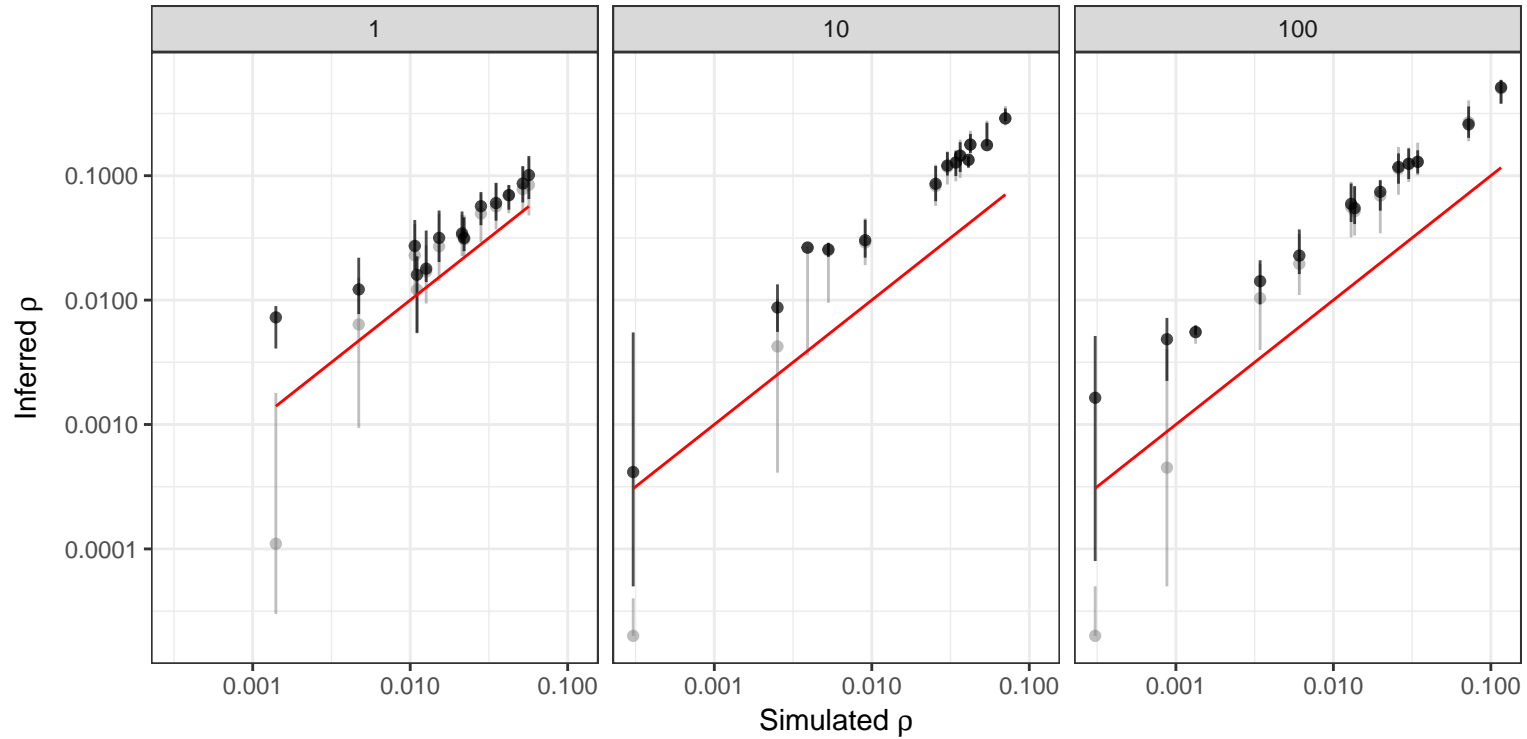

Supplement: Supplementary file 2 [file 1209FigureS2.pdf]

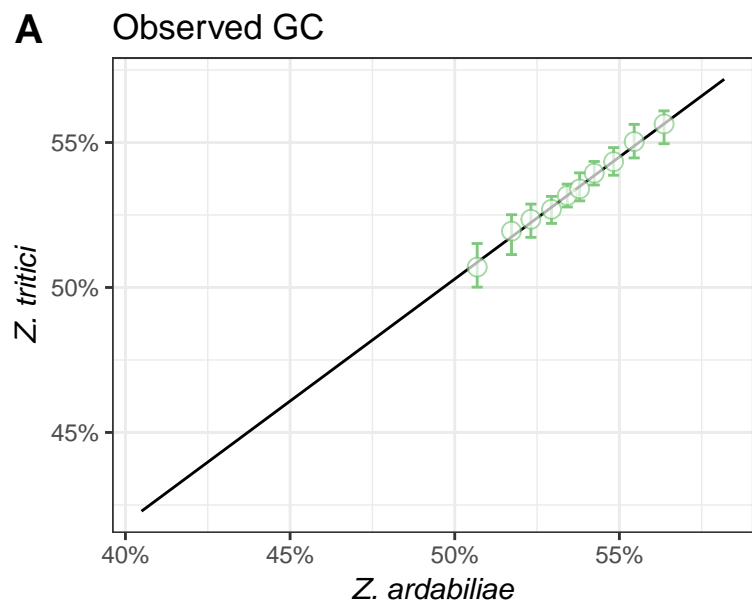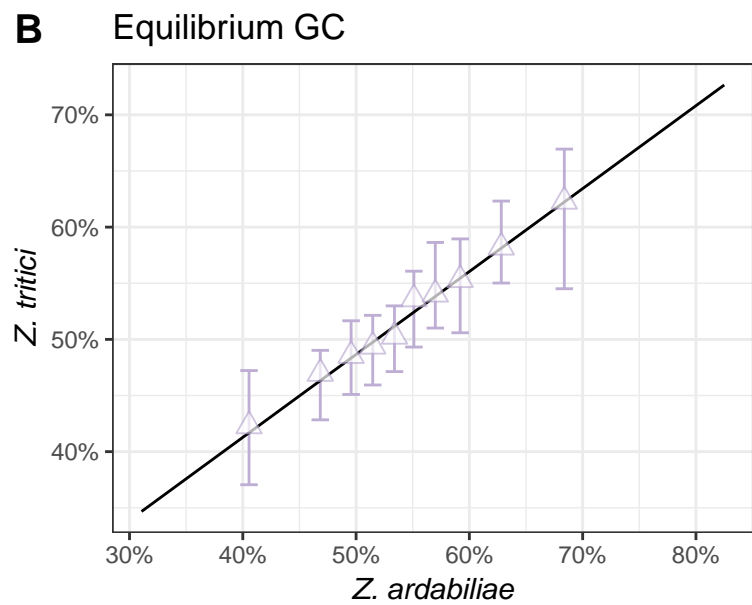

**C**

Type ○ GCobs △ GCeq

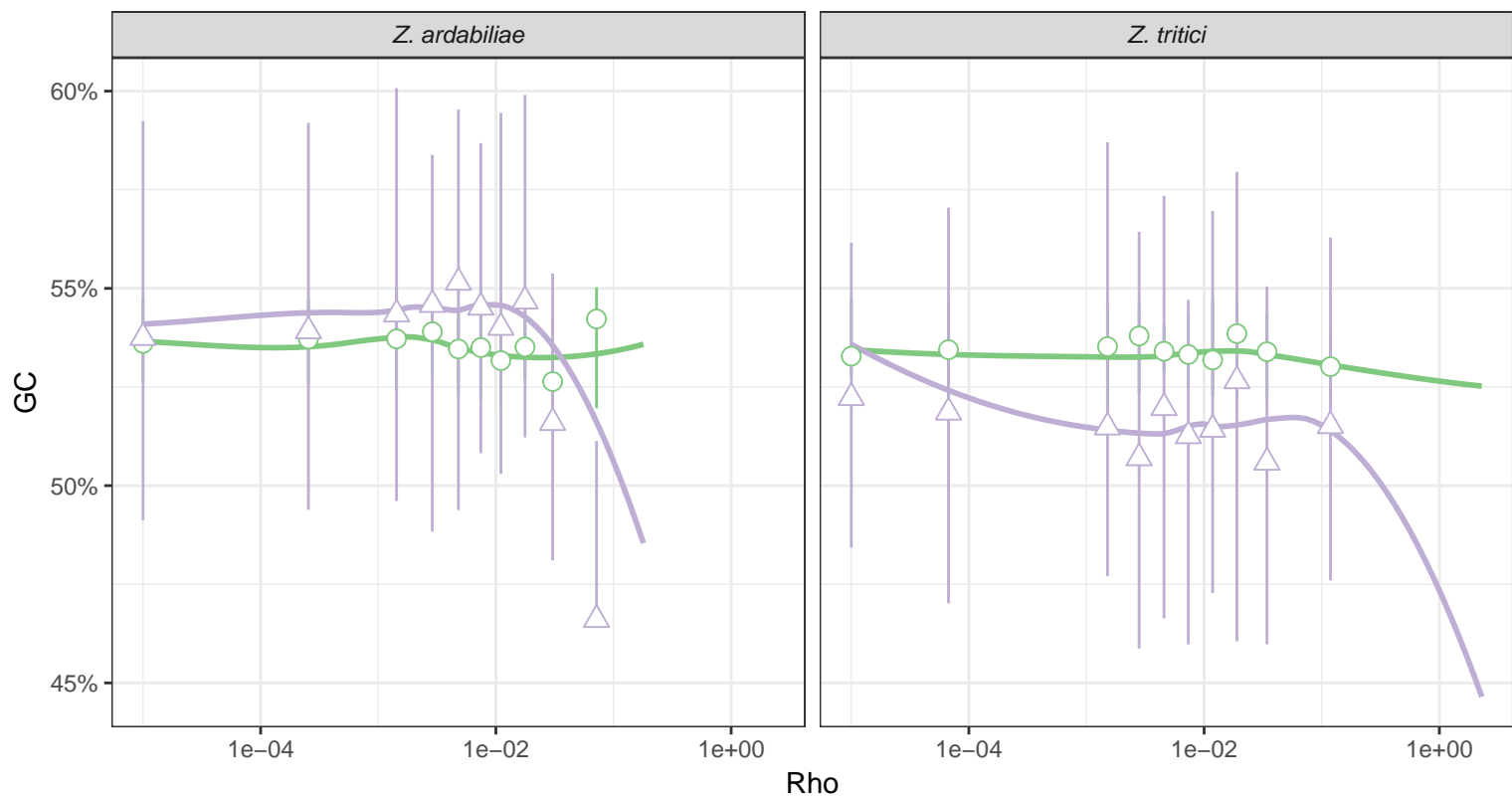

Supplement: Supplementary file 3 [file 1209FigureS3.pdf]

# Chromosome 10

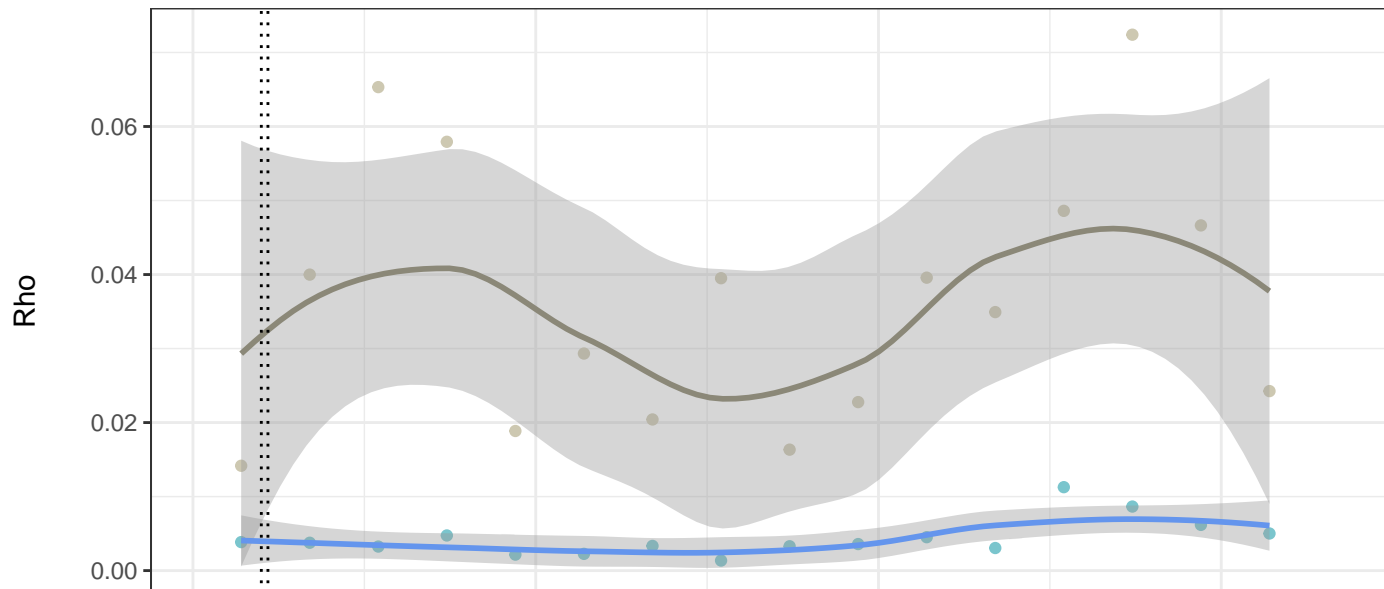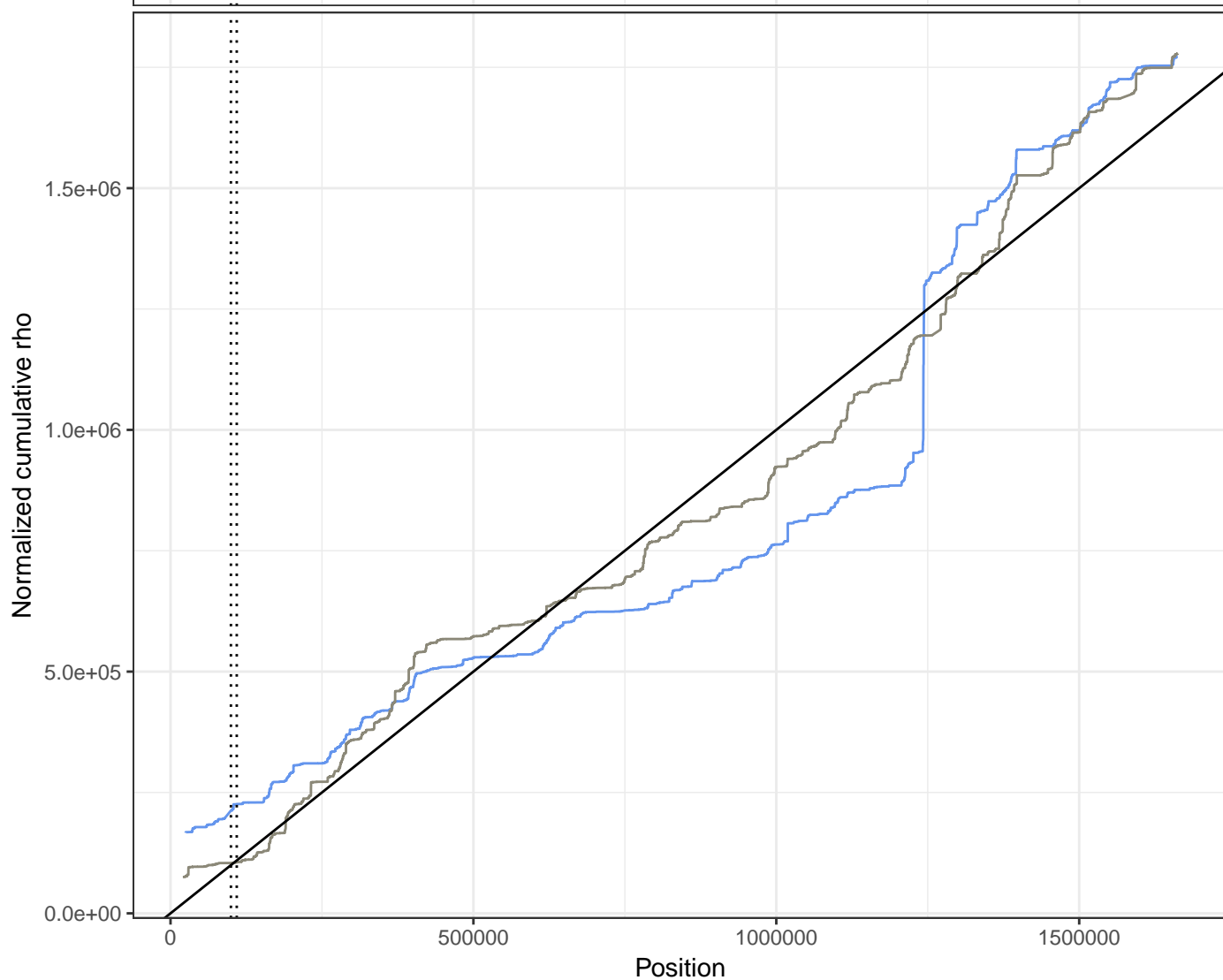

Species — *Z. ardabiliae* — *Z. tritici*

Supplement: Supplementary file 5 [file 1209FileS1.zip › Chromosome10_ZtZa.pdf]

# Chromosome 11

Rho

1.0  
0.5  
0.0

Normalized cumulative rho

1.5e+06  
1.0e+06  
5.0e+05

0

500000

1000000

1500000

Position

Species — Z. ardabiliae — Z. tritici

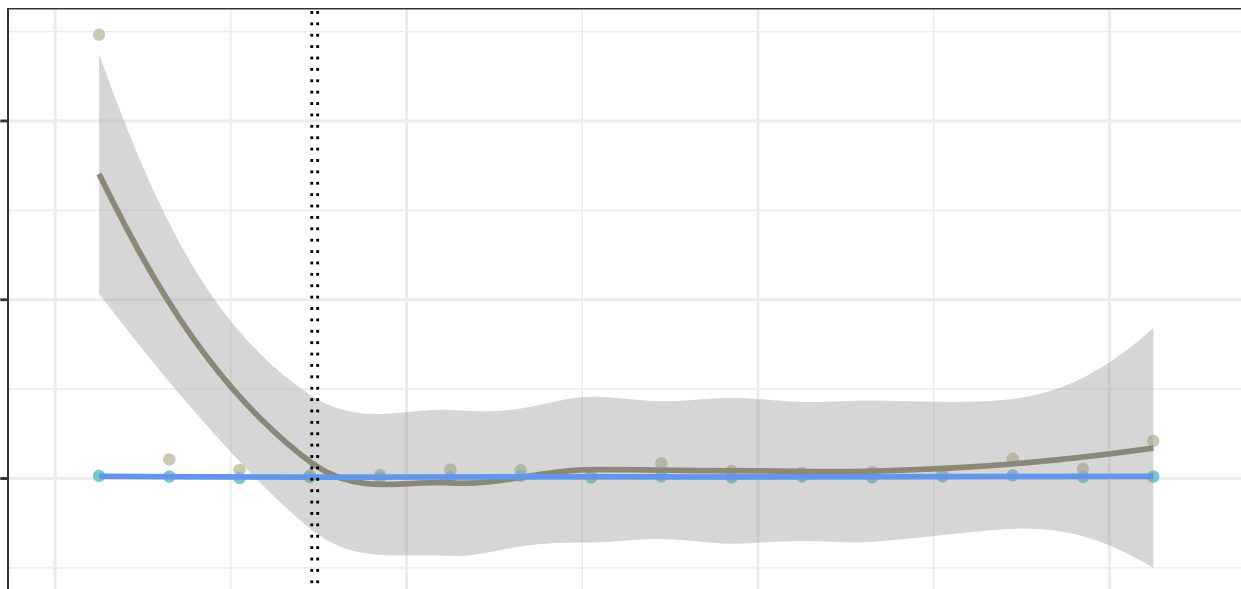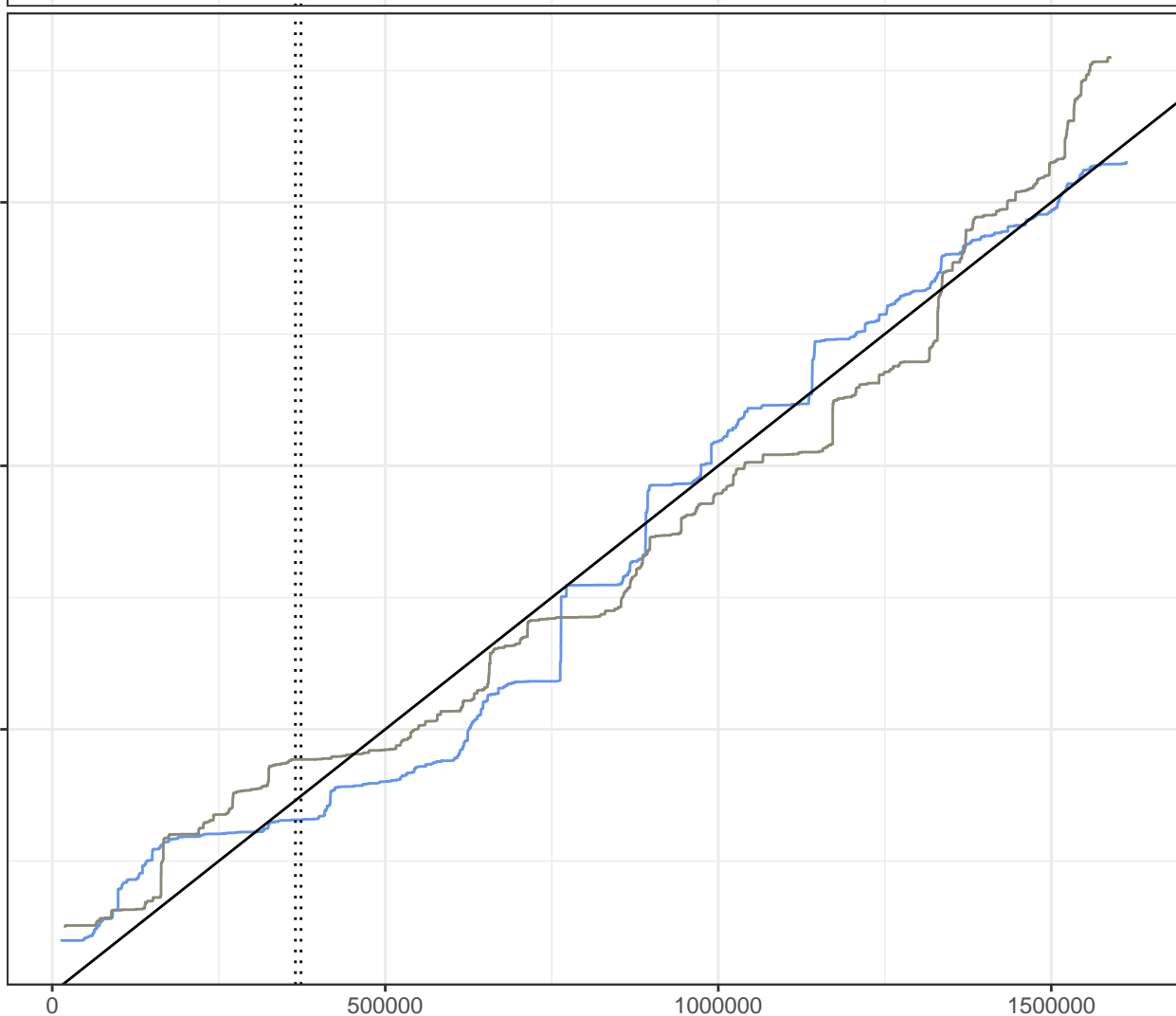

Supplement: Supplementary file 5 [file 1209FileS1.zip › Chromosome11_ZtZa.pdf]

# Chromosome 12

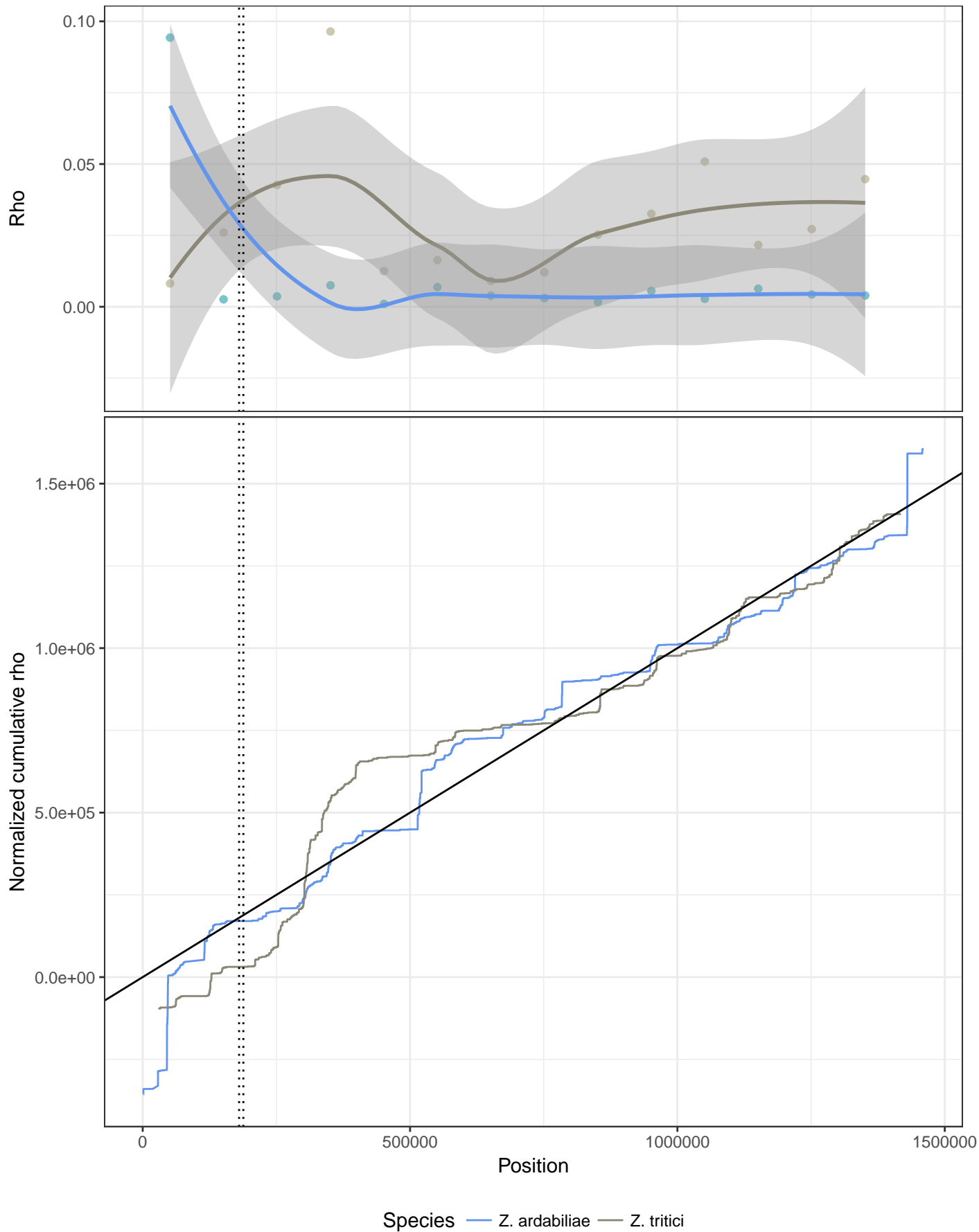

Supplement: Supplementary file 5 [file 1209FileS1.zip › Chromosome12_ZtZa.pdf]

# Chromosome 13

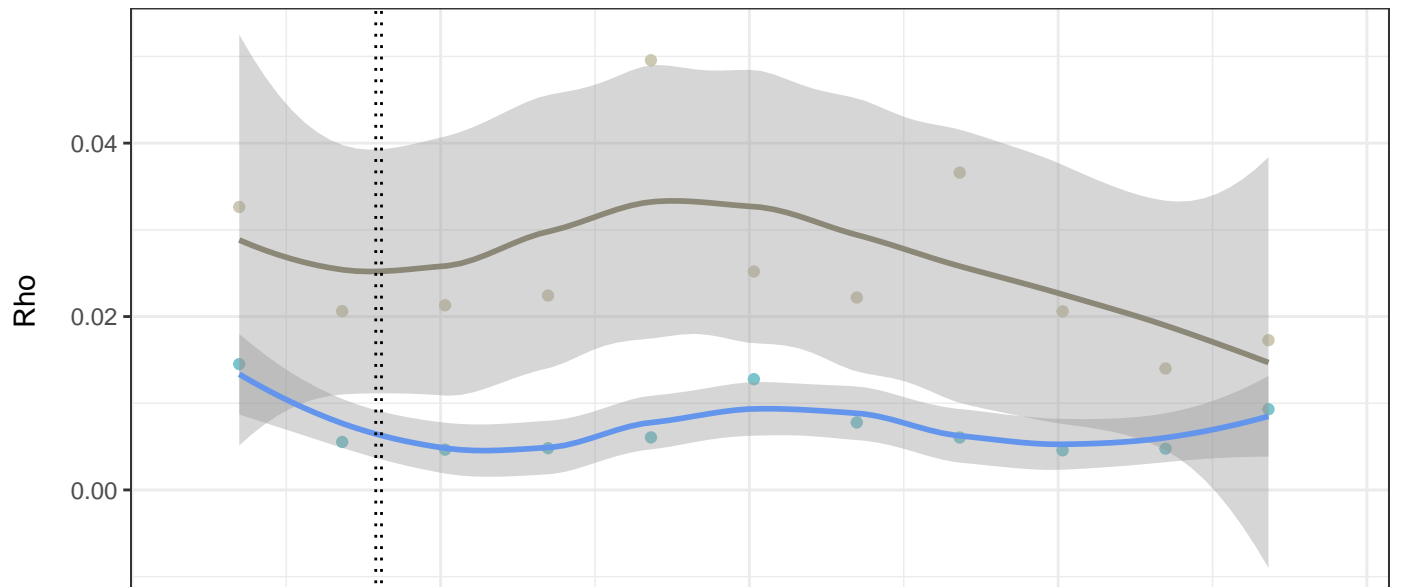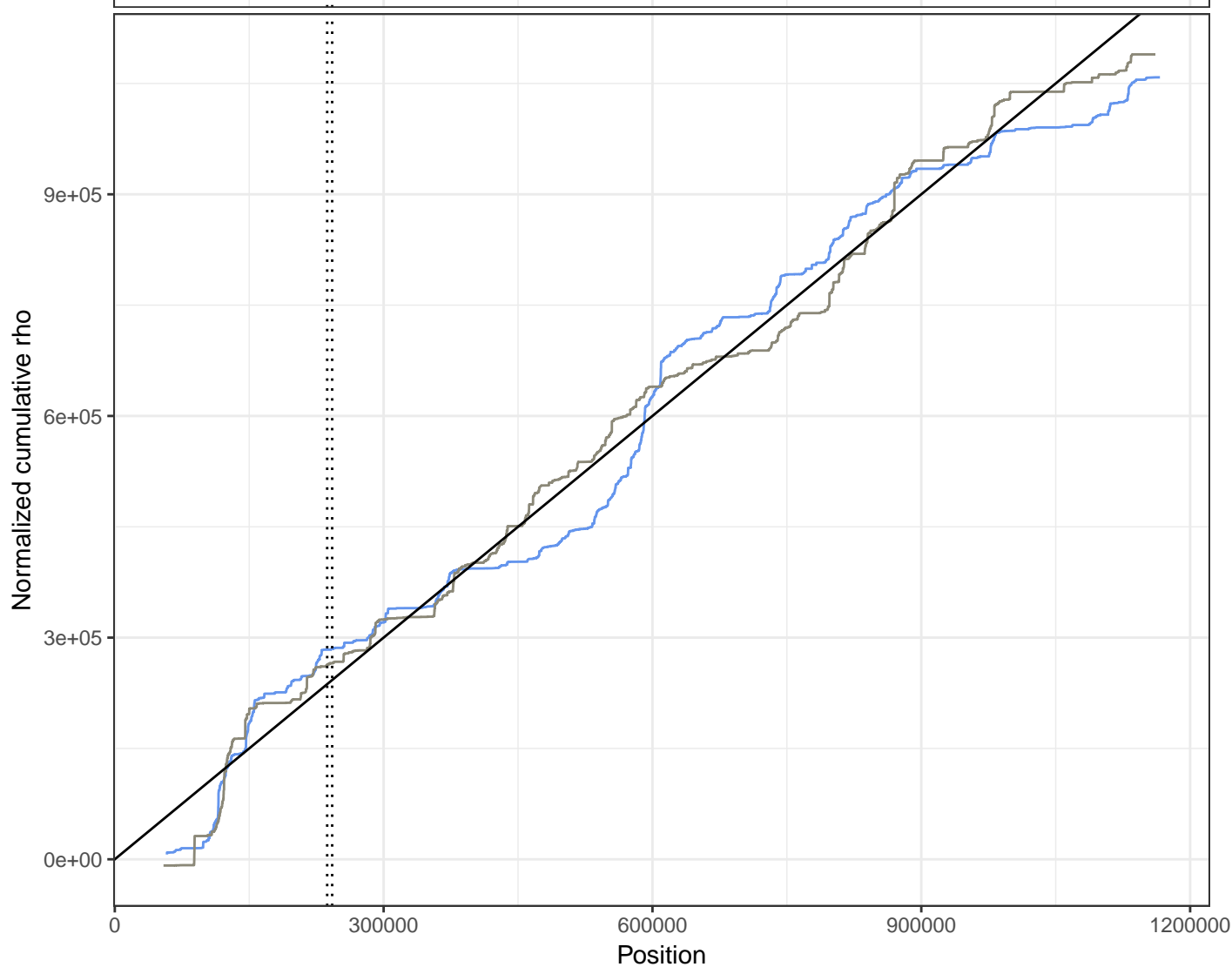

Species — *Z. ardabiliae* — *Z. tritici*

Supplement: Supplementary file 5 [file 1209FileS1.zip › Chromosome13_ZtZa.pdf]

# Chromosome 1

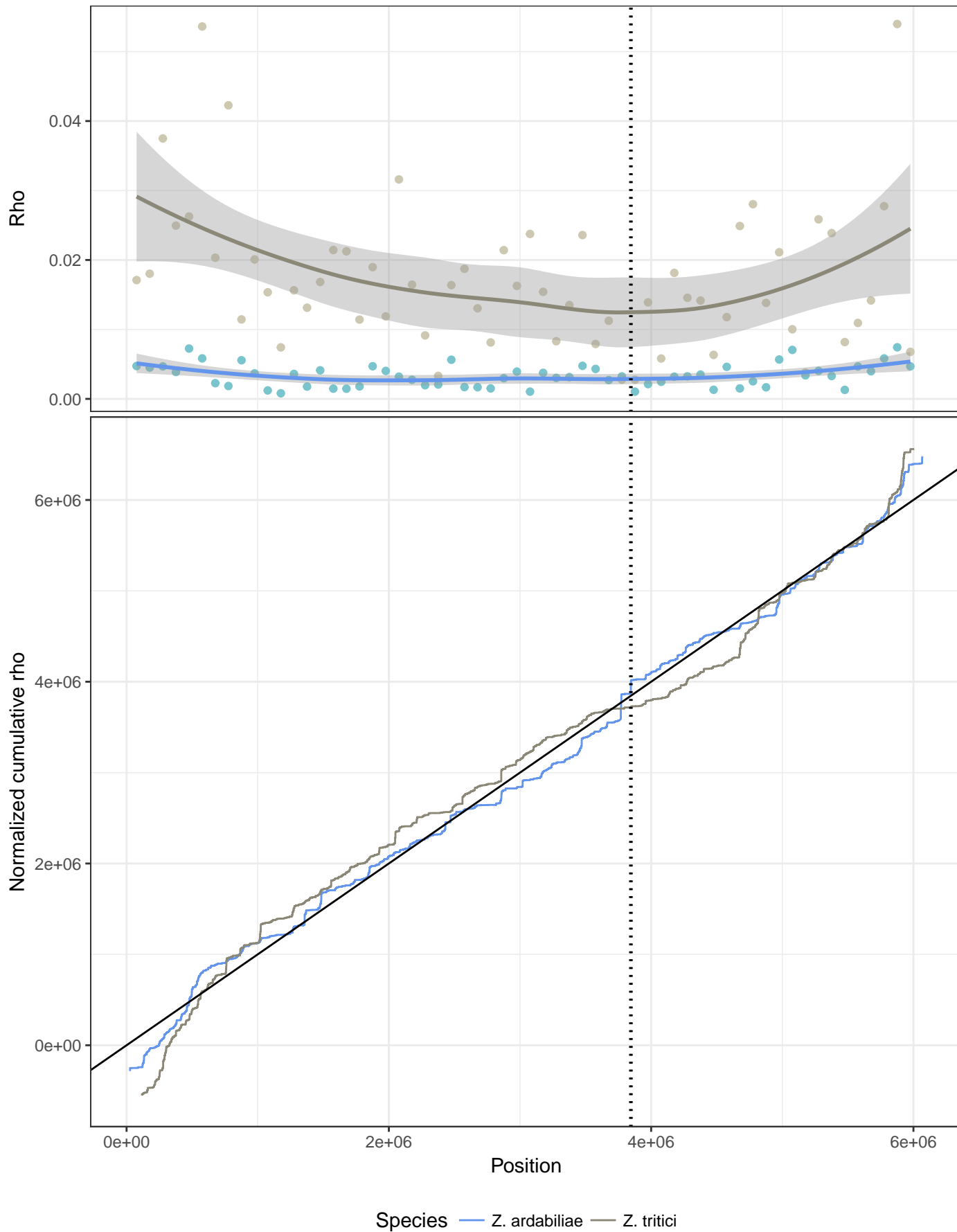

Supplement: Supplementary file 5 [file 1209FileS1.zip › Chromosome1_ZtZa.pdf]

# Chromosome 2

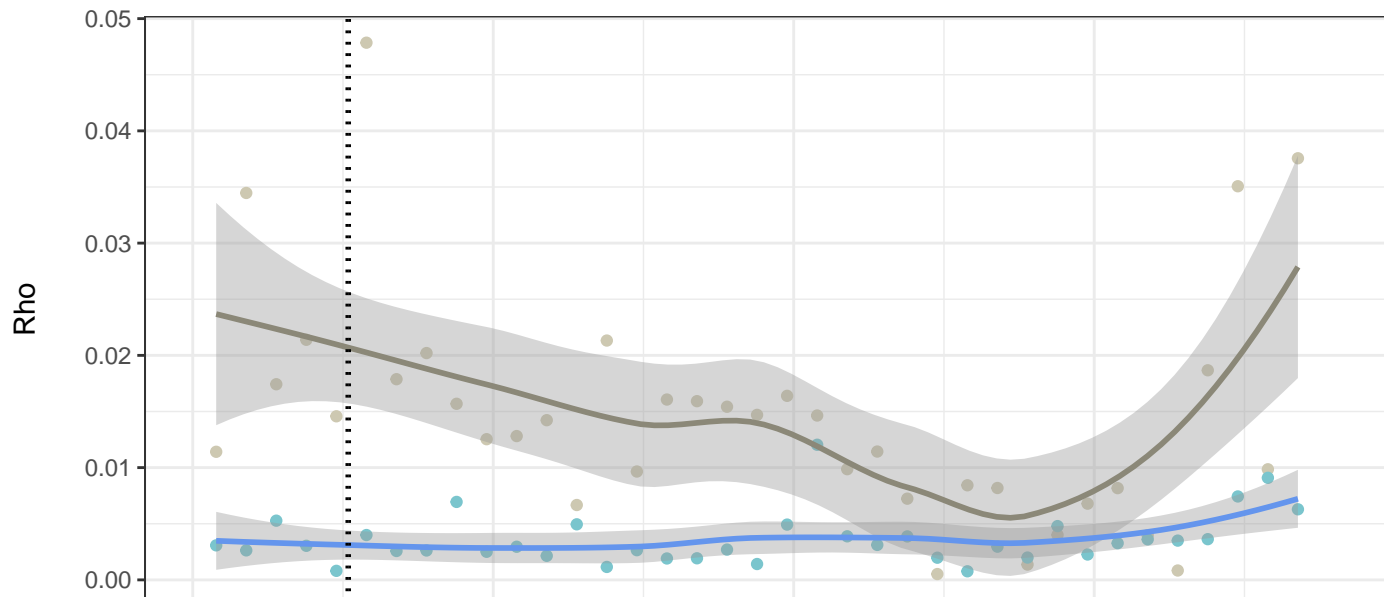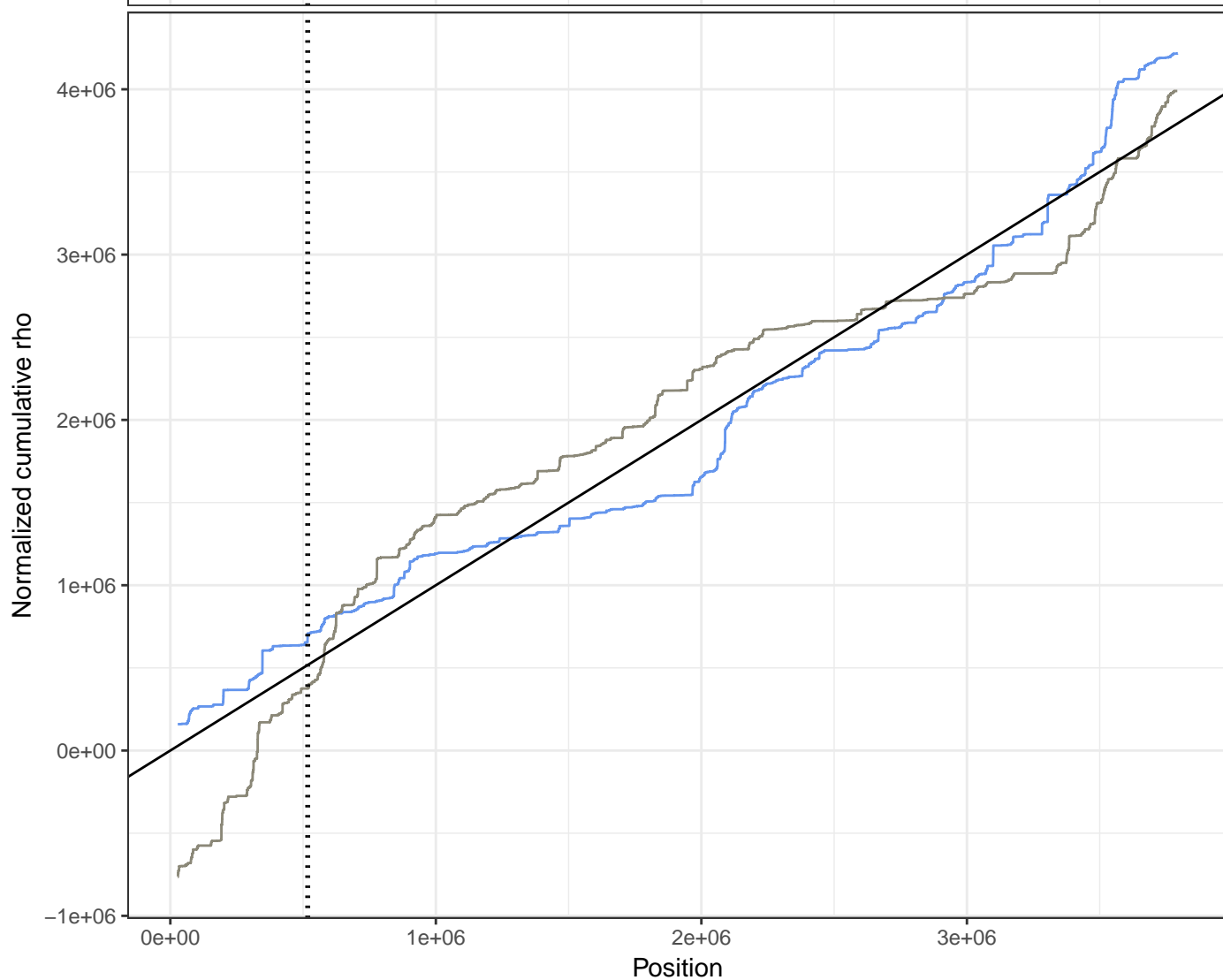

Species — *Z. ardabiliae* — *Z. tritici*

Supplement: Supplementary file 5 [file 1209FileS1.zip › Chromosome2_ZtZa.pdf]

# Chromosome 3

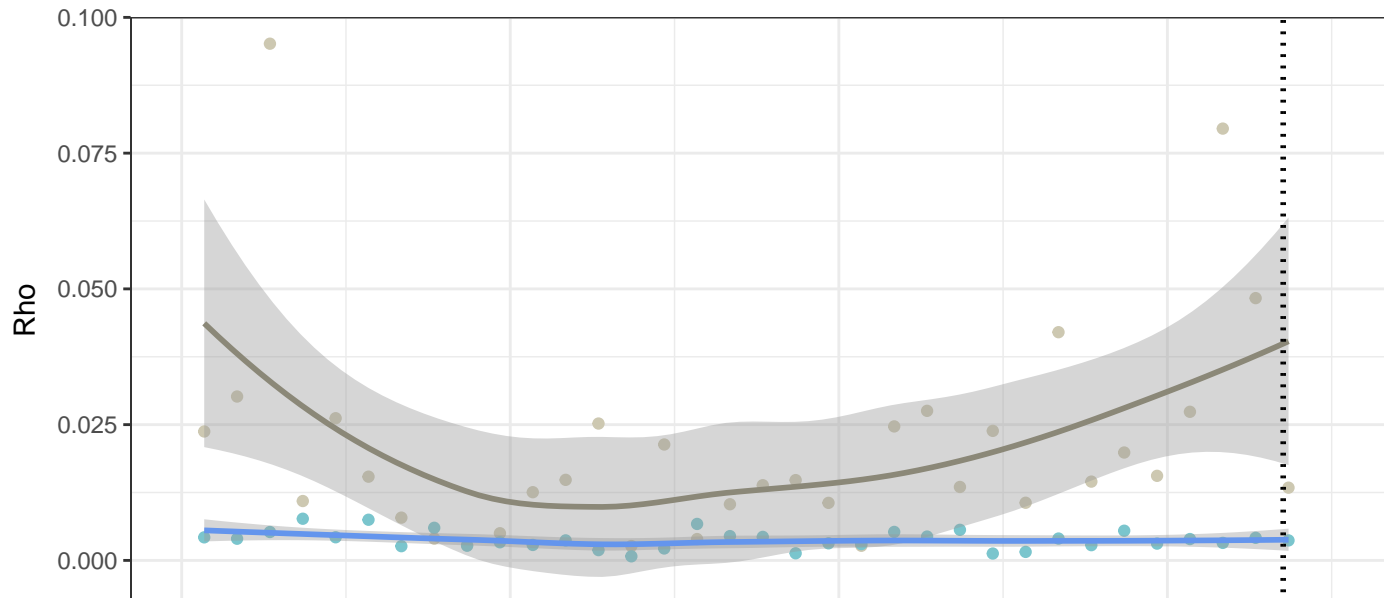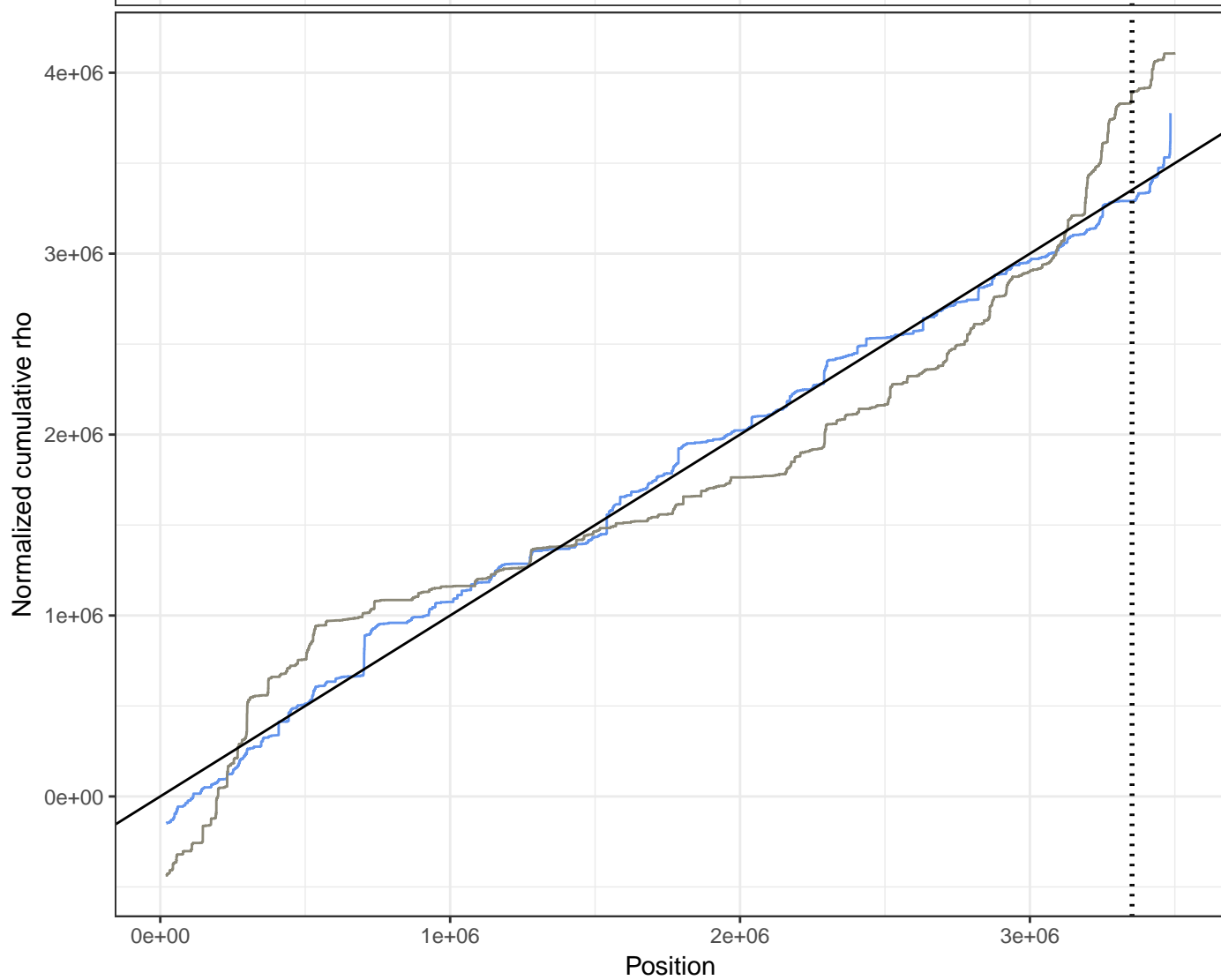

Species — *Z. ardabiliae* — *Z. tritici*

Supplement: Supplementary file 5 [file 1209FileS1.zip › Chromosome3_ZtZa.pdf]

# Chromosome 4

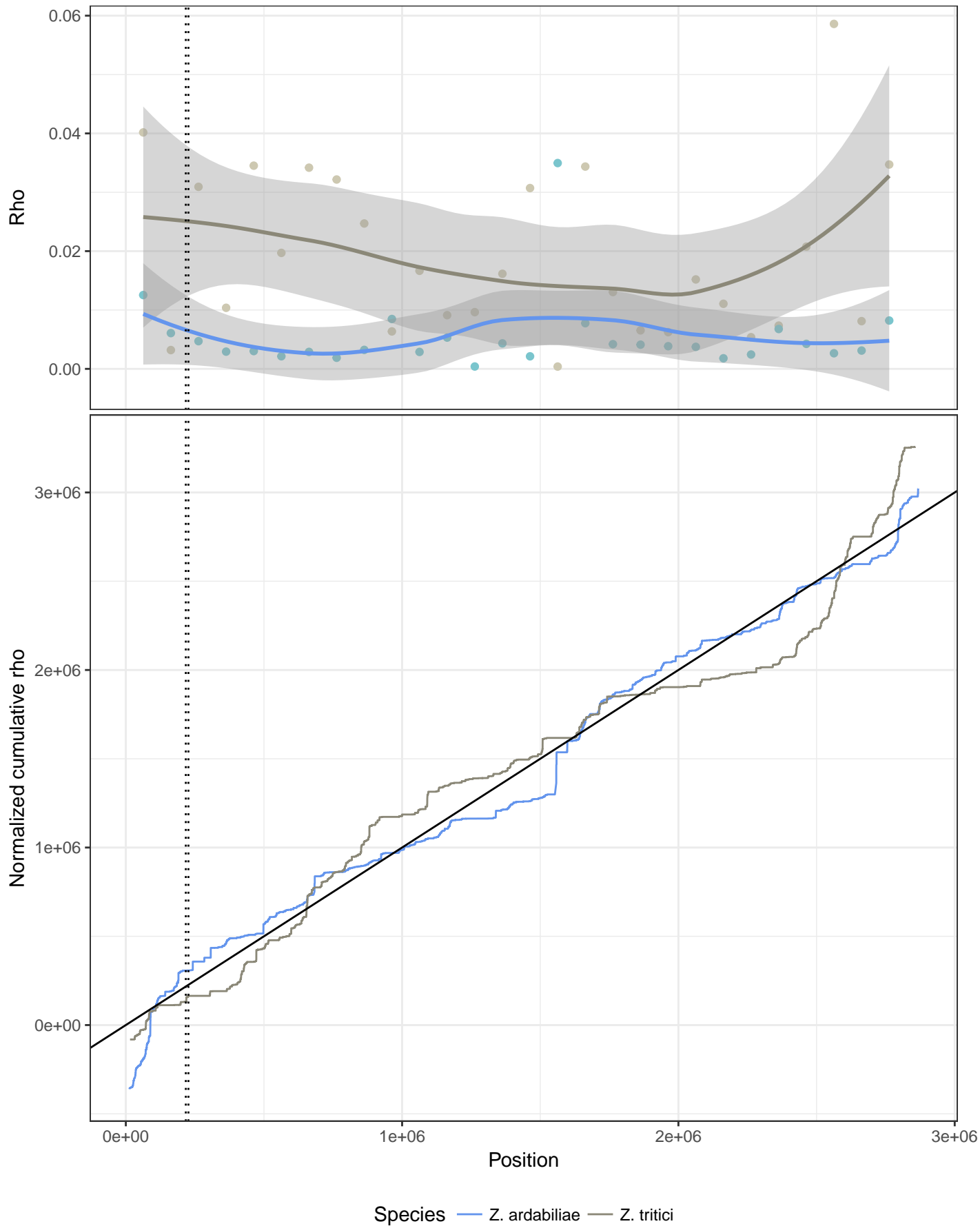

Supplement: Supplementary file 5 [file 1209FileS1.zip › Chromosome4_ZtZa.pdf]

# Chromosome 5

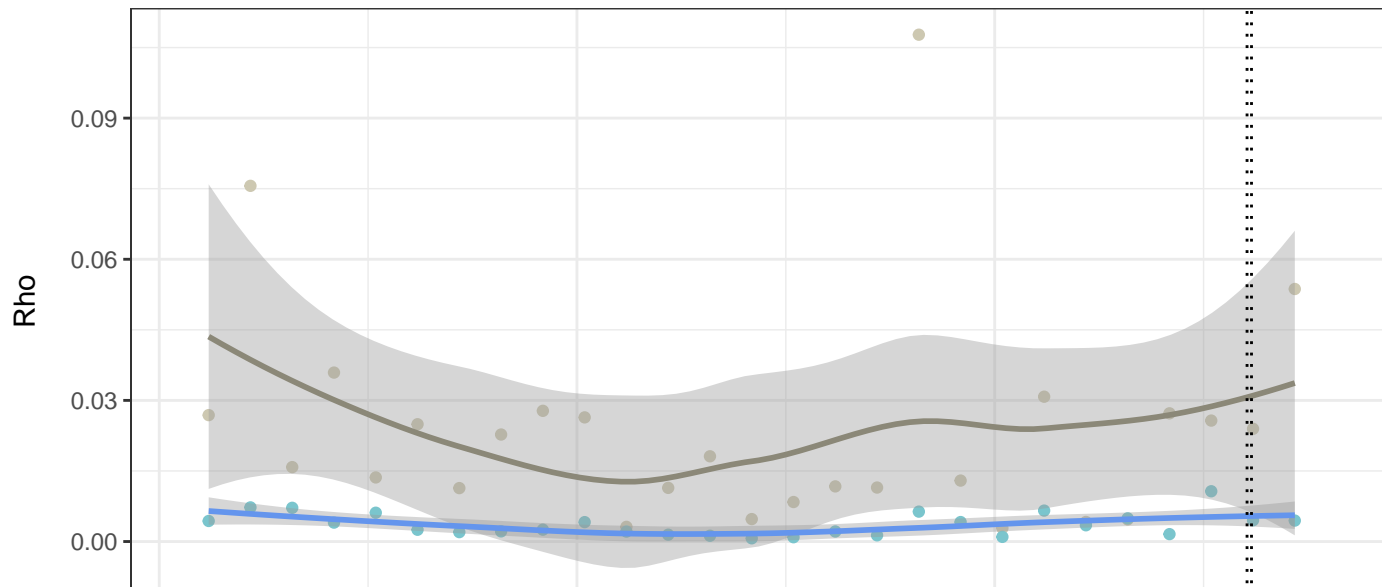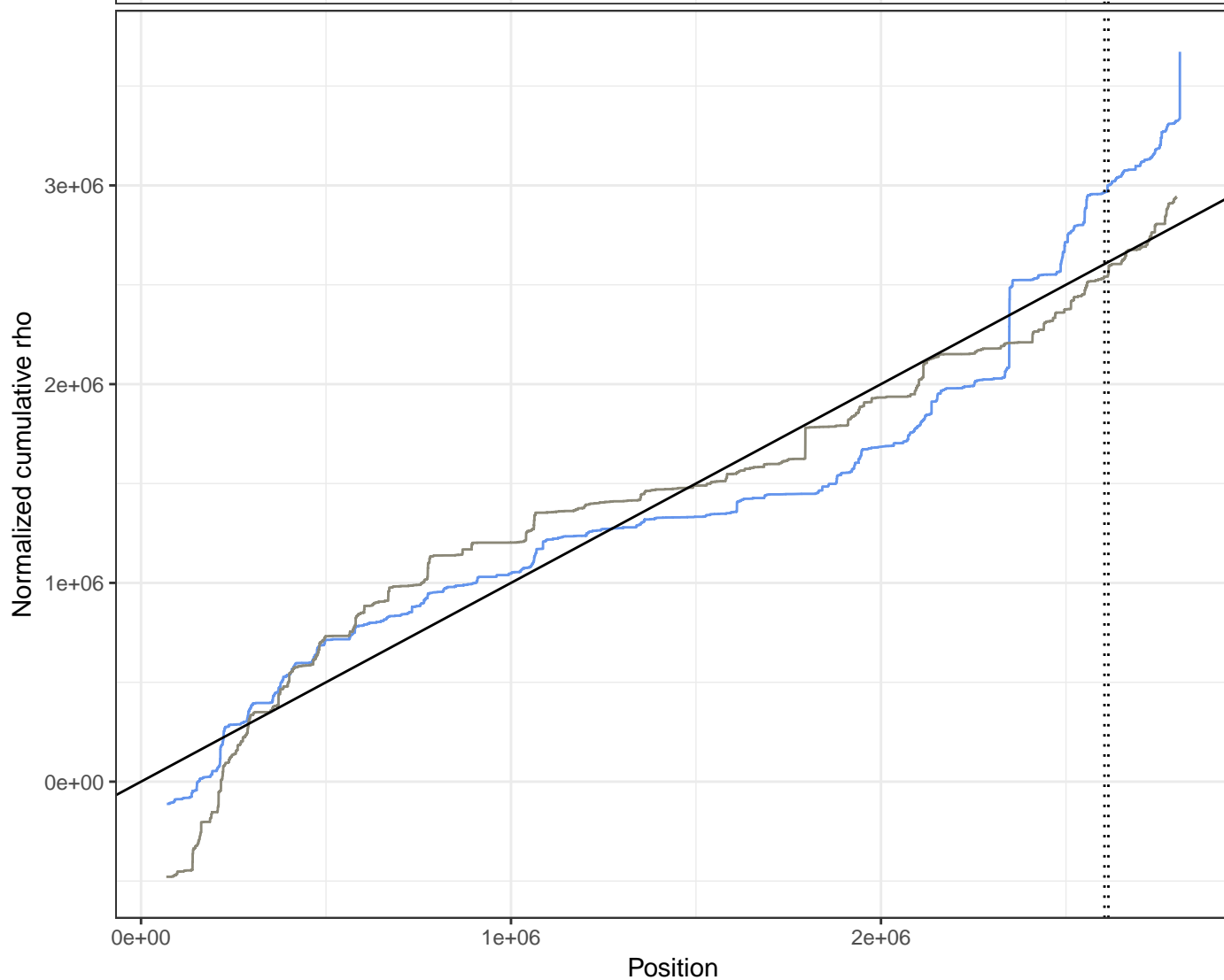

Species — *Z. ardabiliae* — *Z. tritici*

Supplement: Supplementary file 5 [file 1209FileS1.zip › Chromosome5_ZtZa.pdf]

# Chromosome 6

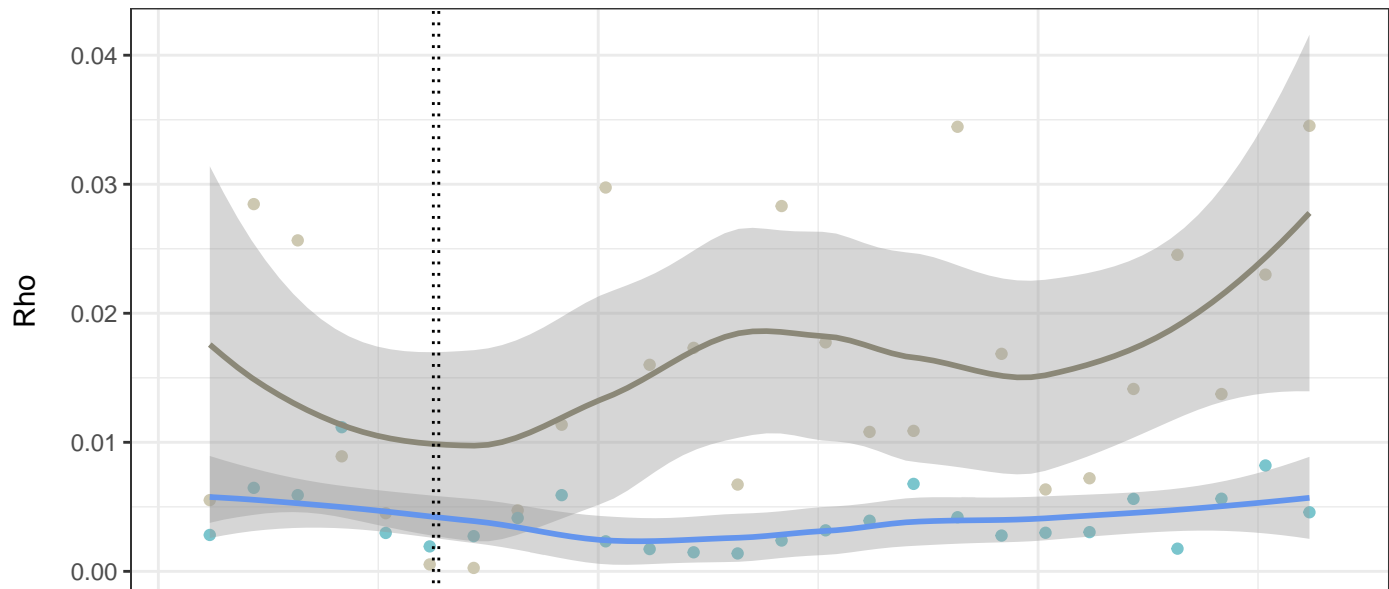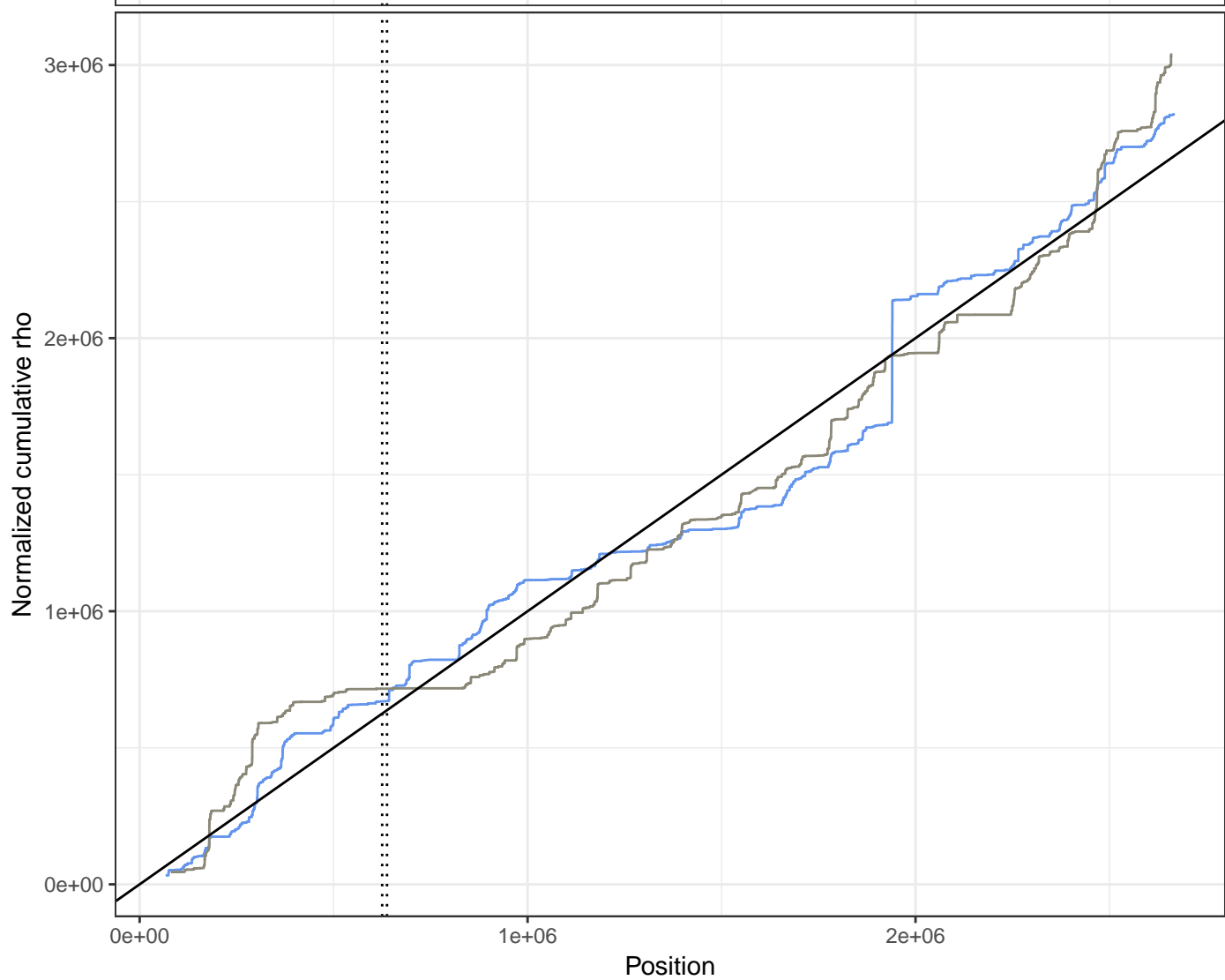

Species — *Z. ardabiliae* — *Z. tritici*

Supplement: Supplementary file 5 [file 1209FileS1.zip › Chromosome6_ZtZa.pdf]

# Chromosome 7

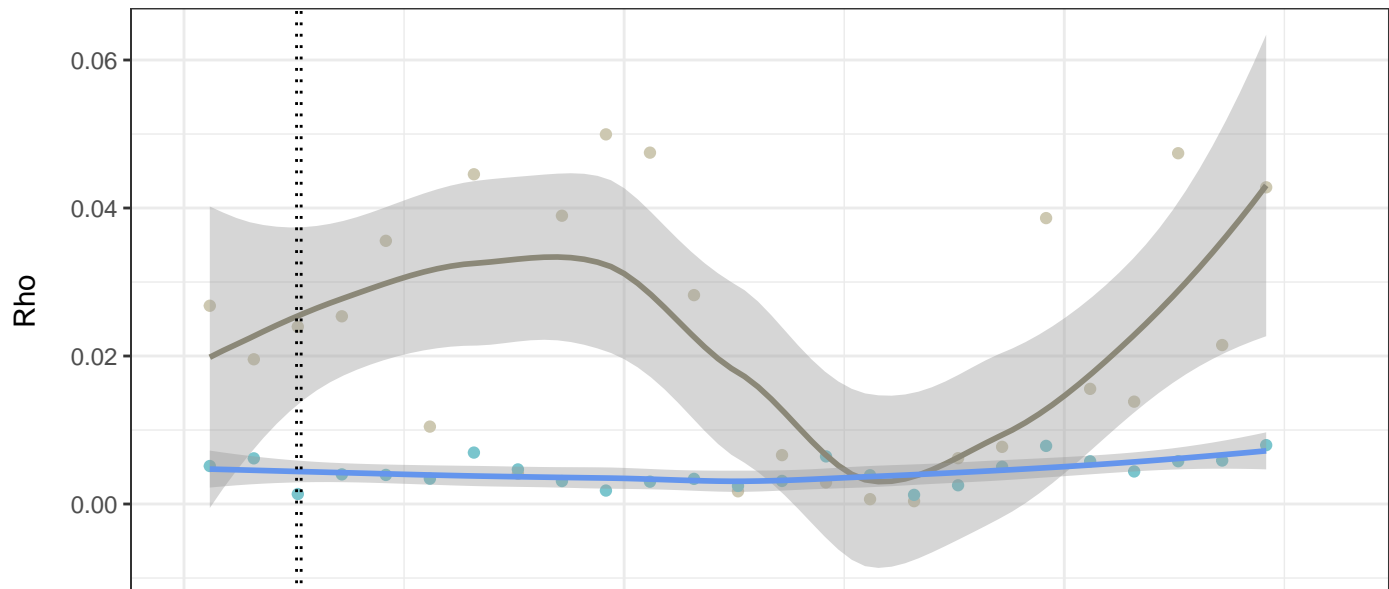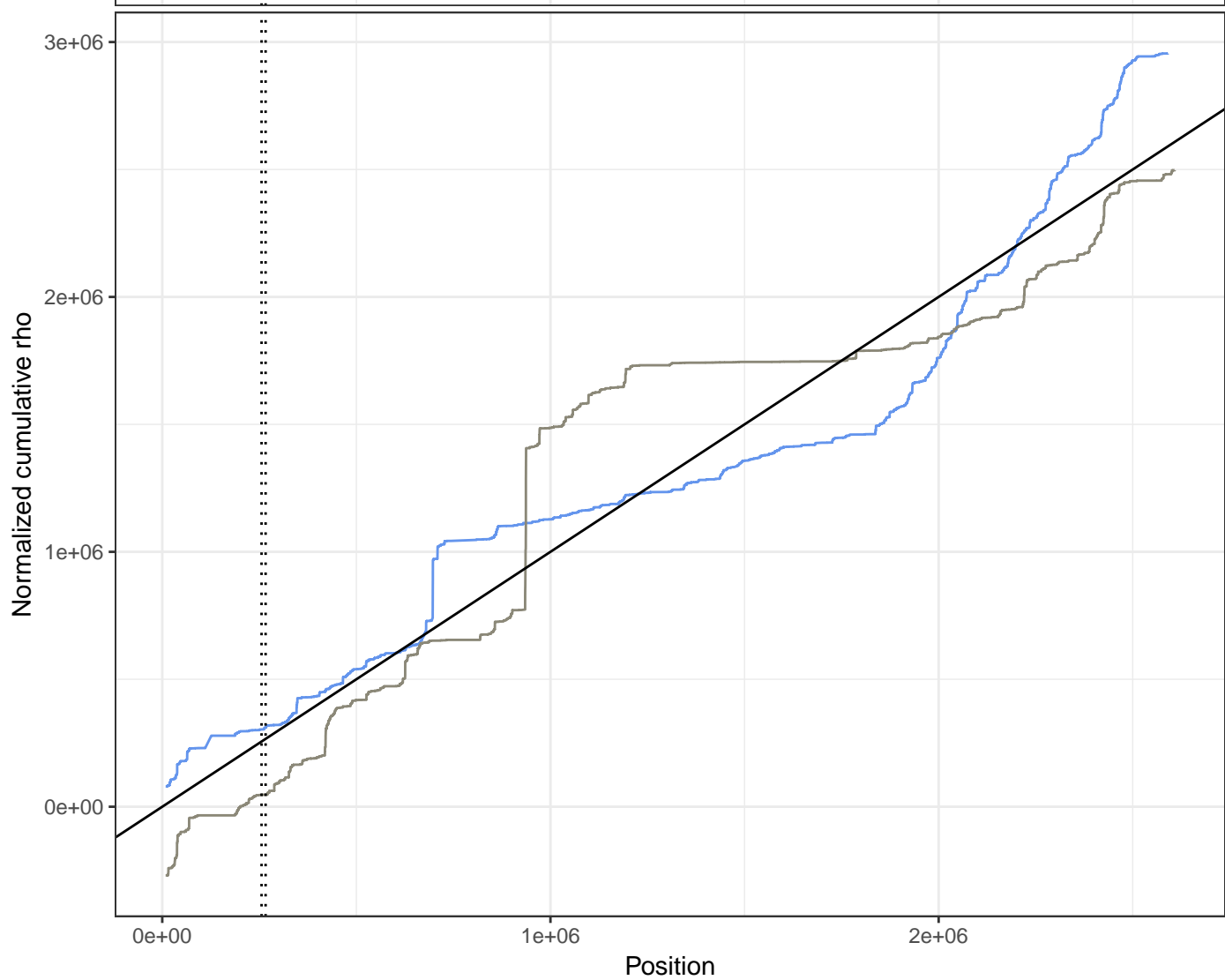

Species — *Z. ardabiliae* — *Z. tritici*

Supplement: Supplementary file 5 [file 1209FileS1.zip › Chromosome7_ZtZa.pdf]

# Chromosome 8

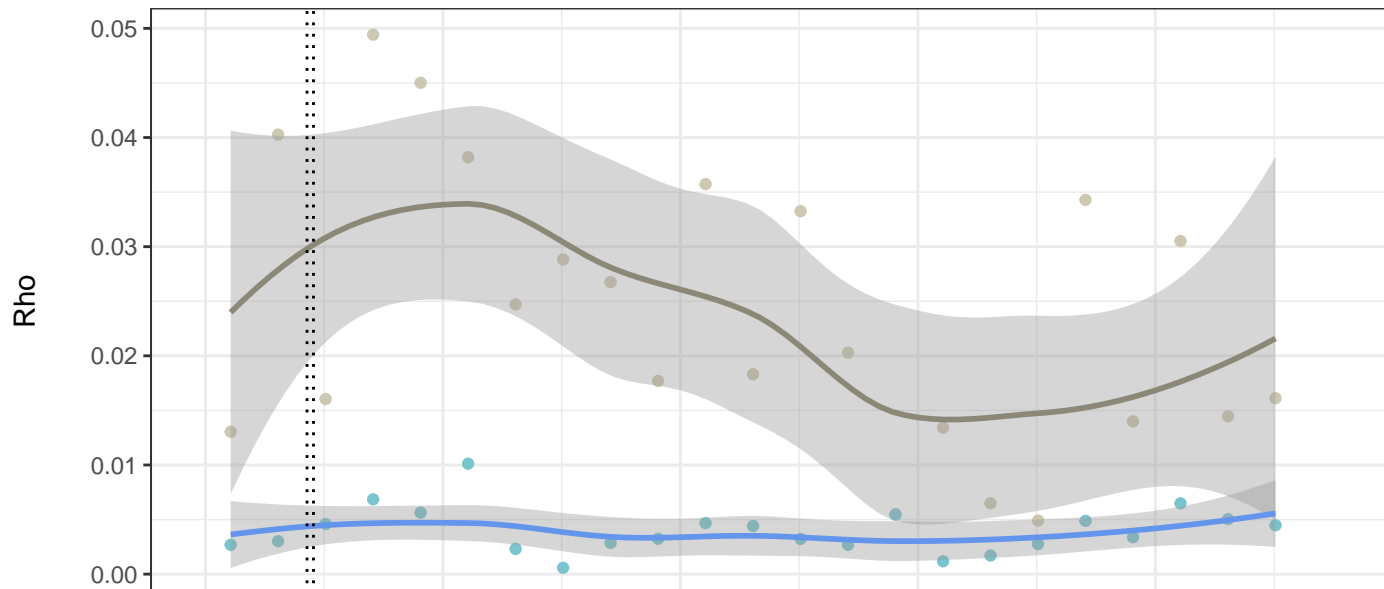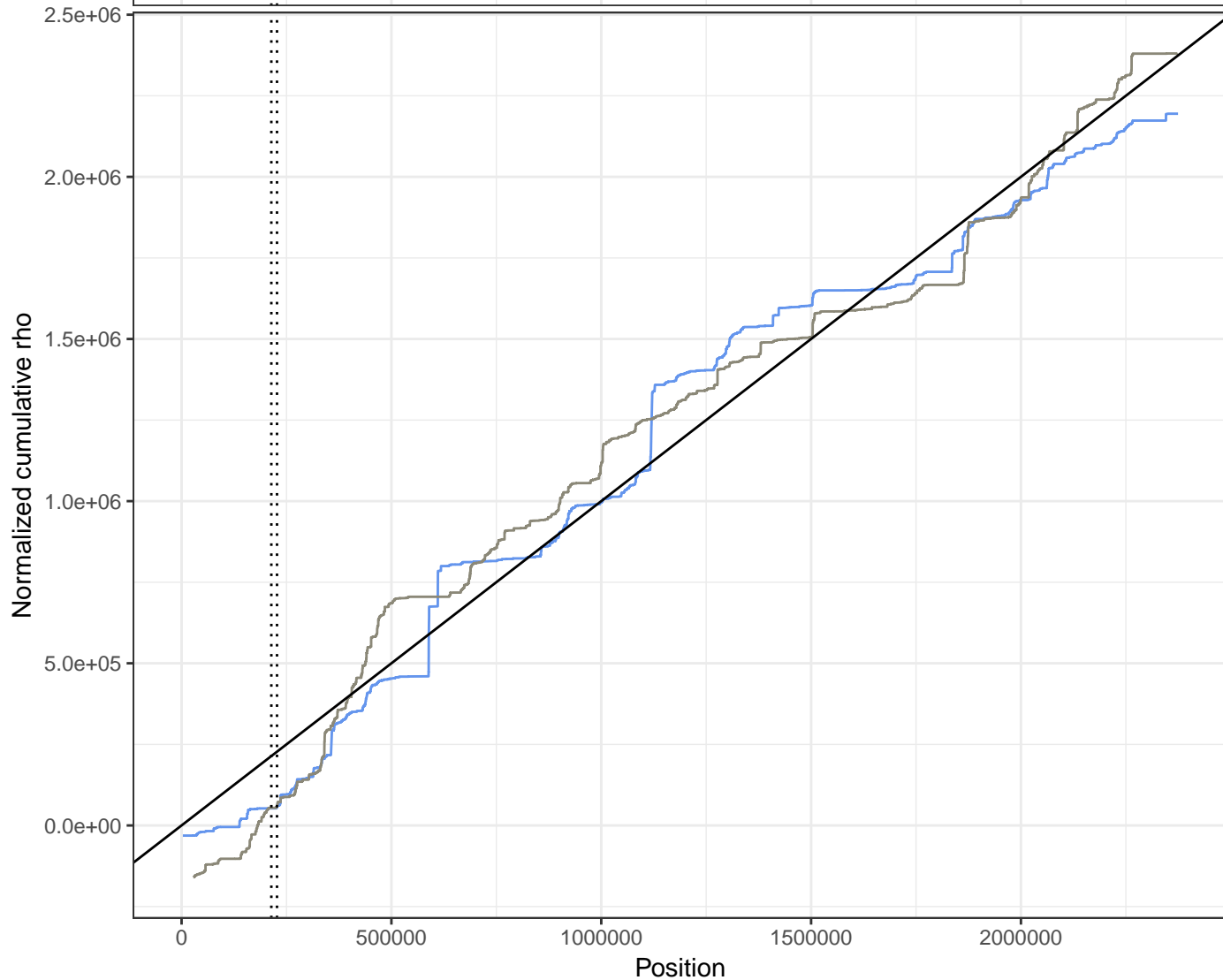

Species — *Z. ardabiliae* — *Z. tritici*

Supplement: Supplementary file 5 [file 1209FileS1.zip › Chromosome8_ZtZa.pdf]

# Chromosome 9

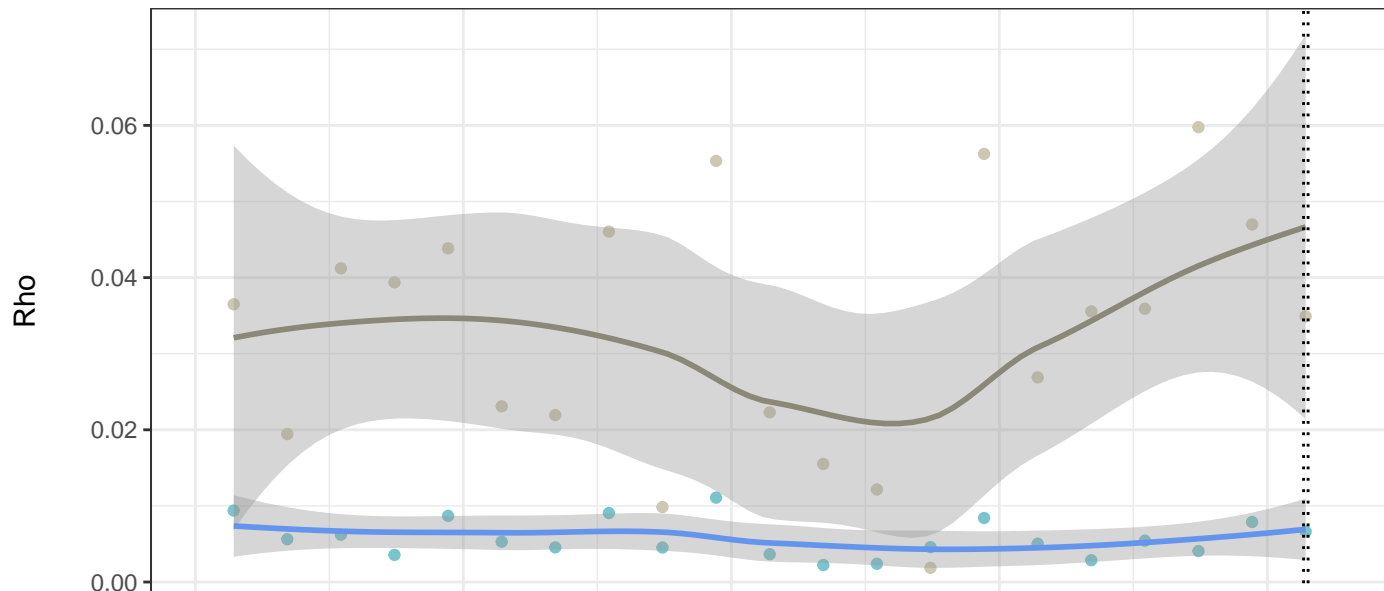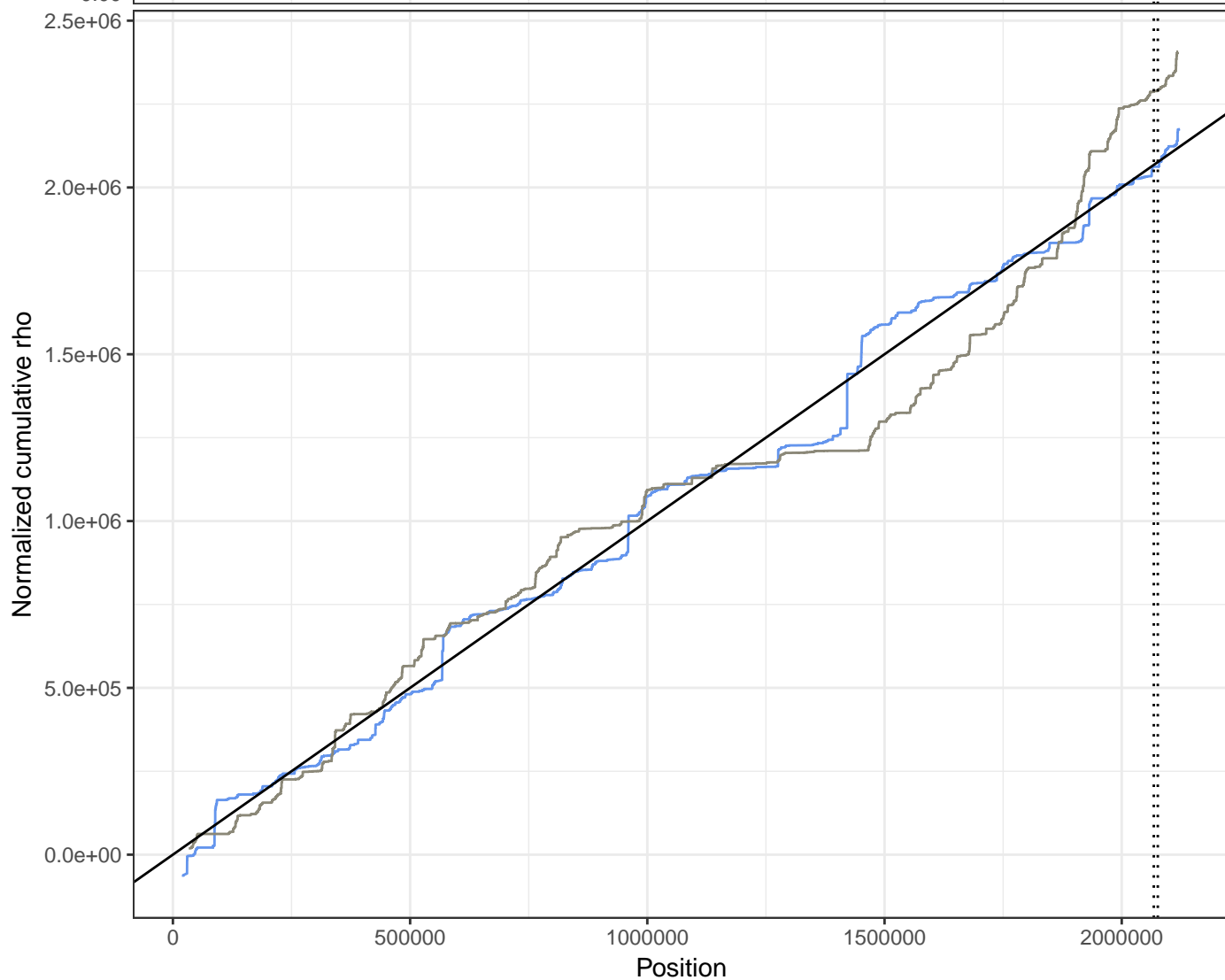

Species — *Z. ardabiliae* — *Z. tritici*

Supplement: Supplementary file 5 [file 1209FileS1.zip › Chromosome9_ZtZa.pdf]
